# Interventions to manage occluded central venous access devices: An umbrella review

**DOI:** 10.1177/11297298241246092

**Published:** 2024-04-24

**Authors:** Jessica Elliott, Linda Ng, Carolyn Meredith, Gordon Mander, Murray Thompson, Lorraine Reynolds

**Affiliations:** 1 School of Nursing and Midwifery, University of Southern Queensland, Ipswich, QLD, Australia; 2 Centre for Health Research, University of Southern Queensland, Ipswich, QLD, Australia; 3 Nursing & Midwifery Education and Training, Darling Downs Health, Toowoomba, QLD, Australia; 4 Faculty of Health and Behavioural Sciences, Southern Queensland Rural Health (SQRH), The University of Queensland, Toowoomba, QLD, Australia; 5 Faculty of Health, School of Clinical Sciences, Queensland University of Technology (QUT), Brisbane, QLD, Australia; 6 Department of Medical Imaging, Toowoomba Hospital, Darling Downs Health, Queensland Health, Toowoomba, QLD, Australia; 7 Medical Workforce, Darling Downs Health, Toowoomba, QLD, Australia

**Keywords:** Catheter occlusion, vascular access devices, catheterisation, peripheral, totally implantable central venous access devices, umbrella review, systematic review

## Abstract

The main objective of this umbrella review is to synthesise available evidence from systematic reviews on the effectiveness of interventions for the management of occlusions in central venous access devices. CVADS have been extensively utilised among the critically ill since the 1950s however have also been linked to an increase in catheter complications. CVAD occlusion can occur in 14%–36% of patients within 1–2 years of catheter placement and is a longstanding complication. Umbrella methodology was applied to review five healthcare databases. Databases were searched for publications from 2009 and 2022 and electronic keywords searches were conducted. The authors searched for reviews that reported on any intervention to prevent, maintain or manage patency of the central venous access devices within an acute care setting. Of the 278 articles identified from the initial search a total of 11 articles were identified. This umbrella review concluded that education enhances patient outcomes and decreases occlusion rates. Further studies are required to explore occlusion reduction strategies in relation to flushing and locking.

## Introduction

Central venous access devices (CVADs) have been extensively utilised in acute care settings since the 1950s and have eased the dilemma of vascular access in many specialities across healthcare.^1–3^ CVADs are catheters that are inserted into central or peripheral veins with the distal tip positioned in the upper right atrium or the distal superior vena cava (SVC).^1^ Totally implanted vascular access devices (TIVADs)/portacaths are a type of CVAD that consist of a small reservoir compartment with a silicone hub for needle insertion and catheter which is tunnelled subcutaneously before entering the central venous system.^1,2^

CVADs are a vital part of management for many medical conditions, providing access for the administration of intravenous medications, fluids, nutrition and blood sampling.^1^ In oncology, these devices have enabled countless treatments and improved the provision of care.^4^ TIVADs provide a secure path to the SVC,^5^ have minimal infectious and thrombotic difficulties compared to external venous catheters^6^ and have low maintenance requirements.^7^

### Background

Although CVADs are regarded as the preferred method in long-term venous access, they have also been linked to an increase in catheter-associated complications.^3^ Complications can be primary, including pneumothorax, air embolism and arterial perforation,^7^ or secondary relating to long-term catheter use. This includes TIVAD reservoir breakdown, occlusion and infection.^8^ In studies investigating impacts associated with catheter complications, infections were attributed to mortality rates of 10%–20% and increased average length of hospital stay.^8^ These problems can disrupt and impede therapy for the primary illness, and negatively impact patient outcomes.^3^

Occlusion occurs in 14%–36% of CVADs within 1–2 years of catheter placement.^6^ Incomplete or partial occlusions occur when blood cannot be withdrawn but fluid can be infused.^8^ Total occlusion occurs when infusion and withdrawal are both not possible.^8^ Occlusion can be mechanical^7^ such as ‘pinch-off syndrome’,^8,9^ chemical^2^ or thrombotic.^10^ Thrombotic occlusion occurs in 66% of adults with a long-term CVAD and can result in long-term vascular problems.^11^ Thrombotic occlusions can result from a fibrin casing (or sheath) surrounding the tip of the catheter^9^ and can occur as early as 24 h of insertion.^11^ Intraluminal clot can occur independently or in combination^8^ and account for 5%–25% of catheter occlusions.^12^

Catheter tip position is a recognised risk factor for occlusions.^12^ Risk increases when the catheter tip terminates in the innominate vein or proximal SVC rather than the distal SVC/right atrial junction.^7,10^

Occlusions are a longstanding complication of CVAD that increase risk of infection, disrupt treatment and have financial implications for the healthcare organisation. For these reasons, early identification and management is vital. Several SRs have reported on interventions to manage CVAD occlusions, however a search of SR repositories (PROSPERO, the Cochrane Database of Systematic Reviews and the JBI Evidence Synthesis journal) did not identify current or ongoing umbrella review on this topic.

## The review

### Objectives

The objective of this umbrella review was to synthesise available evidence to address the following research question: What is the effectiveness of interventions for the management of occlusions in patients with a CVAD?

### Design

This review followed the JBI Umbrella Review methodology guidance.^13^ Reporting of the review was guided by the Preferred Reporting Items for Systematic Review and Meta Analysis (PRISMA) framework.^14^

### Inclusion criteria

The primary outcome of the review was to evaluate catheter occlusions, measured by type, duration and frequency. Occlusion was defined as a blockage that prevents flushing or aspiration of blood from the CVAD.^15^ Reviews that reported on any intervention to prevent, maintain or manage patency of the CVAD in patients aged 18 years or older were included.

Reviews including narrative, brief/rapid and scoping reviews, or those that did not include relevant data on CVAD occlusions were excluded. Studies that did not include full text or were not published in English were also excluded. Where reviews included both paediatric and adult participants, only data pertaining to adult patients were included.

### Search methods

Five electronic databases were searched between 2009 and 2022. The timeframe was chosen as this aligns with the first comprehensive review of management of CVADs undertaken by Cancer Nurses Society of Australia.^16^ The electronic databases used included: CINAHL (via EBSO Host), Cochrane Database of Systematic reviews, EMBASE, JBI Evidence Synthesis Journal, Medline (via OVID), PubMed, Scopus and Web of Sciences. Grey literature was searched using Grey Literature Report and ProQuest Dissertations and Theses.

All potentially relevant articles were imported into EndNote X9 (Clarivate Analytics, PA, USA) for review. The data search was undertaken between the 1st and 30th October 2022.

### Quality appraisal

The quality of the included studies was appraised using the JBI critical appraisal instrument for Systematic reviews and Research Syntheses.^13^ Two reviewers independently appraised each study, with a third reviewer consulted for any conflicts. This umbrella review was registered with PROSPERO [CRD42022382473].

### Data extraction

Data were extracted for review and synthesis (Microsoft Excel). Extracted data included details on study design, participants, sample, settings, follow up and data collection methods. The outcomes, measurement tools and data analysis methods were also extracted. Descriptive and inferential statistics were noted. Findings and discussions were reviewed.

## Results

**Figure 1.**
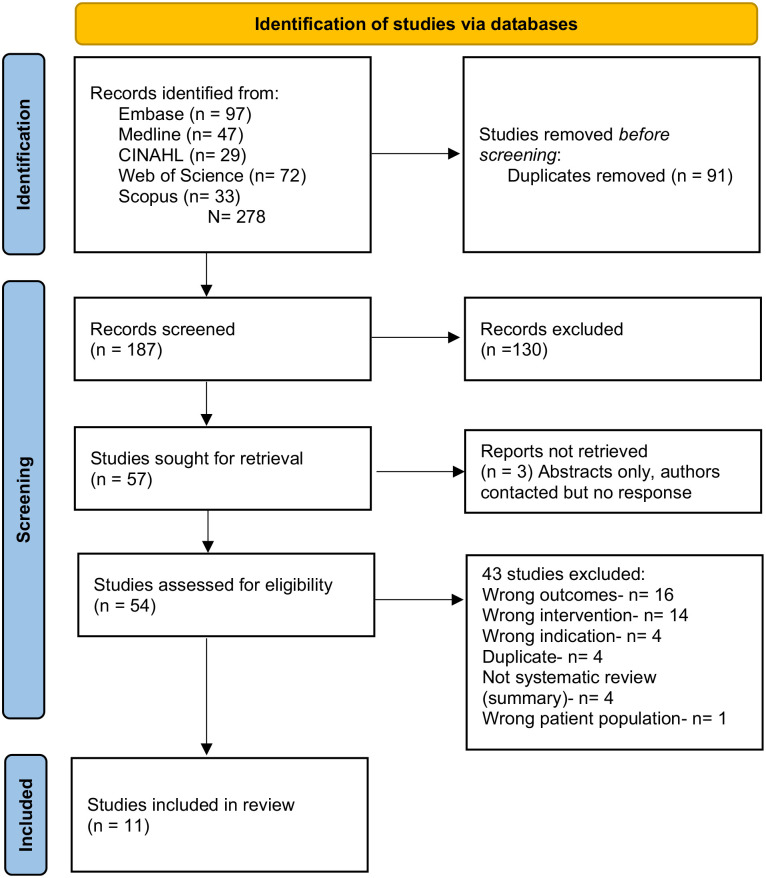
PRISMA. Source: Page et al.^17^

The initial search yielded 278 articles. After removal of duplicates (*n* = 91), the remaining articles were screened with 133 articles excluded. A further 40 articles were excluded following full text examination and a final 11 articles were included in the final review, as detailed in Figure 1 (PRISMA).

### Characteristics of included studies

Of the included articles, there were two each from Italy^18,19^ and Australia,^20,21^ one each from Brazil,^12^ Portugal,^22^ the United States of America^23^ and the United Kingdom,^24^ and three from China.^25–27^ Eleven studies were SRs.^12,18–27^ A summary of review characteristics is displayed in Table 1.

**Table 1.** Summary of characteristics from systematic reviews.

First author (date)	Country	Participants	Intervention(s)	Comparator	Outcome	Included studies	Designs of included studies	Narrative review findings	Critical appraisal tool
Bartock (2010)	USA	Adult patients PICCs	Multiple	Clamped PICCs Location of PICCs	Occlusion rates	9	7 RCTs 2 Expert Opinion/Literature Review/Recommendations	• Dedicated insertion team • Use of valved PICCs • Nurse education	Johns Hopkins Nursing Evidence-Based Practice Rating Scale
Clari et al. (2021)	Italy	Adult patients TIVADs	Prolonged flushing and locking intervals	Short flushing/locking	Occlusion rate	6	Observational (cohort and case-control)	• No statistically significant difference between locking and flushing intervals	ROBINS-I Cochrane RoB tool
Dal Molin et al. (2014)	Italy	Adult patients CVCs	Heparin	Other substances	Occlusion	8	RCTs	• Evidence of no difference in effectiveness vs saline • Unclear effectiveness of other solutions vs heparin	Critical Appraisal Skills Programme (CASP)
da Costa et al. (2018)	Brazil	Adult cancer patients CVCs	Multiple	NA	Restored patency	15	13 RCTs 2 Observational	• Evidence for restoration with thrombolytic therapy • Significant RoB and high clinical and statistical heterogeneity limits findings	Cochrane MINORS RoB
Ferreira Dos Santos et al. (2015)	Portugal	Adult patients CVCs	Heparin	Versus 0.9% saline solution	Occlusion rate	9	8 RCTs 1 Observational	• No significant difference between groups	Grid for critical evaluation of an article describing a prospective, randomised and controlled clinical study, Centro de Estudos de Medicina Baseada na Evidência da Faculdade de Medicina de Lisboa, Portugal JBI Critical Appraisal Checklist for Cohort and Case-control studies
Pan et al. (2019)	China	Adult patients PICCs	Nursing interventions	Standard care	Occlusion rate	13	9 RCTs 4 quasi-experimental	• Nurse education effective • Evidence to support valved PICCs • Heparin effective • Pulsatile flushing effective	Cochrane criteria for RCTs and quasi-experimental studies
van Miert et al. (2012)	UK	All patients CVADs	Multiple	Multiple	Efficacy and safety to restore patency	8 (7*)	RCTs	• Limited evidence to support Urokinase • Insufficient evidence to conclude efficacy and safety for drug interventions	Cochrane RoB tool
Seckold et al. (2015)	Australia	Adult patients PICCs	Silicone PICC lines	Polyurethane PICC lines	Complication rates (all causes)	21	1 RCT 1 quasi-experimental 18 Observational	• Complication rates similar between groups	JBI critical appraisal tool
Slaughter et al. (2020)	Australia	Adult patients CVCs or PICCs	Impact of material and design	Standard of care	Thrombosis and secondary endpoints	9	RCTs	• No significant difference between catheter types	Cochrane RoB tool
Xu and Zhang (2022)	China	Adult patients PICCs	Heparin Concentrations	NA	Thrombosis and secondary endpoints	7	2 RCTs 5 quasi-experimental	• Increased concentrations significantly affect coagulation indicators and occlusion rates	Unclear
Zheng et al. (2019)	China	Adult patients CVADs	Multiple	NA	Restoring patency	7	1 RCT 2 Observational	• Restoring patency from lipid deposition with sodium hydroxide effective • Quality of evidence is poor	JBI Critical Appraisal Tool

NA: not available; CVCs: central venous catheters; PICCs: peripherally inserted central catheters; TIVADs: totally implanted vascular access devices; RoB: risk of bias.

* Included duplicate published report.

Two studies reviewed staff education programmes,^23,25^ two studies reviewed the impact of different CVAD types for example, anti-thrombotic on risk for complications^21,24^ and 10 studies looked at the impact of various solutions versus standard saline solution for flushing and locking.^12,18–20,22,26,27^ A summary of findings from Meta-analyses is reported in Table 2.

**Table 2.** Summary of findings from Meta-analyses.

Review author (date)	Intervention	Comparator	Outcome	Sample size (*n*)	No. of included studies	Effect size metric	Meta-analysis statistical model	Summary effect estimate	95% CI	Heterogeneity
Pan et al. (2019)	Nursing Education	Standard Care	Occlusion Rate	799	3 studies	Risk ratio	Fixed effects (Mantel-Haenszel) model	0.31	0.19–0.51	*I*^2^ = 0.00%
	Valved PICCs	Non-valved PICCs	Catheter Occlusion	599	5	Risk ratio	Fixed effects (Mantel-Haenszel) model	0.60	0.32–1.15	*I*^2^ = 0.00%
Clari et al. (2021)	Prolonged flushing and locking intervals	Short flushing and locking	Occlusion prevention	880	4	Relative risk No effect	Fixed effects (Mantel-Haenszel) model	0.81	0.41–1.61	*I*^2^ = 0.00% (*p* = 0.69)
da Costa et al. (2018)	All forms of thrombolytic therapy	Various	Restored patency	888	14	Proportion	Random effects model	0.841	0.76–0.90	*I*^2^ = 86.37% (high)
Dal Molin et al. (2014)	Heparin	Sodium Chloride	Restored patency	1685	5	Median log Odds Ratio	Random effects model (network meta-analysis)	0.55	0.12–1.37	Cochran’s Q
van Miert et al. (2012)	Urokinase	Placebo	Restored patency	287	2	Risk ratio	Fixed effects (Mantel-Haenszel) model	2.09	1.47–2.95	*I*^2^ = 0.00%
Ferreira Dos Santos et al. (2015)	Heparin	Sodium Chloride	Risk of occlusion	1866	7	Risk ratio	Random effects (Mantel-Haenzel) model	0.68	0.41–1.10	*I*^2^ = 9%
Slaughter et al. (2020)	Anti-thrombolytic catheters	Standard catheters	Thrombosis	1894	8	Risk ratio No effect	Random effects model	0.98	0.87–1.11	*I*^2^ = 0.00%
Xu and Zhang (2022)	100 U/mL Heparin	50 U/mL Heparin	Catheter Occlusion rate	266	3	Odds ratio	Fixed effects (Mantel-Haenzel) model	0.48	0.24–0.96	*I*^2^ = 29%
Zheng et al. (2019)	Sodium hydroxide and ethanol	Various	Restored patency	168	6	Proportion	Random effects model	0.77	0.55–0.92	*I*^2^ = 84%

### Education methods

Two reviews^23,25^ compared the efficacy of nursing education in reducing CVAD occlusion rates. In a review by Bartlock,^23^ the efficacy of education in reducing the rate of CVAD occlusion was explored through additional training sessions for nursing staff and efficacy of a dedicated team with ‘intensive training’ in PICC management. Bartlock’s^23^ review included a total of 1621 participants, with two studies excluding sample size information. The review by Bartlock noted a reduction in occlusions from both interventions.

Pan et al.,^25^ (*n* = 13; 9 RCTs, 4 quasi-experimental; *n* = 1398) identified three quasi-experimental studies that involved nursing education as an intervention for reducing PICC occlusions in oncology patients. Education aimed to improve the capability in PICC insertion and aftercare.^25^ In all three primary studies, the incidence of occlusion decreased following nursing education. The outcome of the included meta-analysis revealed that providing nurses with education in PICC management significantly reduced the incidence of occlusion relative to standard care (Relative Risk (RR): 0.31, 95% confidence interval (CI) [0.19, 0.51]). Both reviews^23,25^ concluded education and training for nursing staff appeared to have an overall positive effect on minimising CVAD-related complications however limitations were noted based on methodological heterogeneity.

Based on the findings by both Bartlock^23^ and Pan et al.,^25^ education and training for nursing staff appeared to have an overall positive effect on minimising CVAD-related complications like occlusions however there were limitations based on heterogenicity and how outcomes were measured.

### Central venous access device types

Two SRs^20,25^ compared different types of CVADs and their effectiveness at reducing occlusions. Pan et al.,^25^ included studies that compared different PICC valve and locking mechanisms. It was concluded that the incidence of occlusion was not reduced by valved PICCs in the experimental group (RR: 0.60, 95% CI [0.32, 1.15]).

Seckold et al.^20^ (*n* = 21; 1 RCT, 1 quasi-experimental study, 18 observational studies; *n* = 4693) compared silicone versus polyurethane PICCs and post-insertion complications. Many included studies involved silicone PICCs followed by polyurethane PICCs. Findings from these reviews suggest that PICC type or material did not seem to have a significant impact on minimising post insertion complications including occlusion. No statistical significance was calculated for this. Specific population groups such as oncology and medical/surgical were reported to have on average, higher rates of post-insertion complications (oncology: 33.2%, medical/surgical: 22.3%).

The results from the studies by Pan et al.,^25^ and Seckold et al.,^20^ suggest that peripherally inserted central catheter type or material did not seem to have a significant impact on minimising post insertion complications including occlusion.

### Flushing and locking CVADs

Most SRs focused on the efficacy of various substances at reducing the risk of insertion complications. Pan et al.,^25^ (*n* = 13; 9 RCTs, 4 quasi-experimental; *n* = 1398) identified three studies that investigated the effect of flushing. Significant methodological heterogeneity due to variations in the solutions used between the studies meant that they could not be meta-analysed.

Clari et al.,^18^ reviewed five retrospective and one prospective studies (*n* = 2135 participants) involving CVADs flushed and locked with heparin solutions of varying concentrations. The majority of participants were female and had a diagnosis of cancer. No significant difference was identified between prolonged and short flushing intervals.

da Costa et al.^12^ reviewed 15 articles (13 clinical trials, 2 observational studies) to evaluate the efficacy and safety of various substances at restoring catheter patency when used at varying concentrations and intervals. The results showed that catheter restoration time varied. An overall restoration rate of 0.841, 95% CI [0.76, 0.90] was identified. A subgroup meta-analysis by drug type was also conducted. Overall, the most common intervention used to treat thrombotic catheter occlusions were urokinase and alteplase.

Dal Molin et al.^19^ (8 RCTs, *n* = 1821) reported the effectiveness of heparin against other solutions. The review identified four studies comparing concentrations of heparin with sodium chloride, and heparin in varying concentrations compared to urokinase (*n* = 2), vitamin C (*n* = 1) and lepirudin (*n* = 1). Concentration and frequency of flushes with solutions varied across studies. The review concluded that there was evidence that heparin is no more effective than flushing with sodium chloride.

Ferreira Dos Santos et al.^22^ (*n* = 9; 8 RCTs, 1 cohort study) reviewed the effectiveness of heparin compared to 0.9% sodium chloride for maintaining permeability in various types of CVAD. As part of the studies, double lumen (*n* = 2), triple lumen (*n* = 2), multi lumen (*n* = 1), fully deployed (*n* = 2), peripherally inserted central catheter and central venous catheter (CVC) (*n* = 2) catheters were used. Generally, most studies concluded that the standard saline solution was effective in maintaining CVC permeability. The summary estimate provided by the meta-analysis showed no statistical significance. However, it did show that there was an increased risk of non-permeable CVCs in the saline group (RR: 0.68, 95% CI [0.41, 1.10], *p* = 0.12), hence, a beneficial effect in the heparinised flush group.

van Miert et al.^24^ reviewed seven RCTs (*n* = 632) to compare the efficacy and safety of different interventions to restore patency of occluded CVC lumens in both adult and paediatric populations. No studies investigated the restoration of patency using chemical or surgical interventions however, the RCTs reviewed the comparisons of different thrombolytic drugs for treating CVC occlusion thought to be caused by a thrombus. Overall, the authors reported there was inadequate evidence to determine the efficacy and safety of individual drug interventions for restoring catheter patency.

Slaughter et al.^21^ (*n* = 9; 8 RCTs, 1 pilot study; *n* = 2061) reviewed the effectiveness and safety of anti-thrombogenic materials and alterations to CVC design on thrombosis rates. Of these studies, three specifically discussed changes involving anti-thrombotic or anti-clotting coating CVCs. The meta-analysis indicated no statistically significant difference between anti-thrombogenic coated catheters and uncoated catheters (RR: 0.98, 95% CI [0.87, 1.11]). There was no significant difference between the experimental and control groups (RR: 0.77, 95% CI [0.23, 2.61]). The review concluded that due to the small sample size, primary study quality and heterogeneity, it was not appropriate to draw firm conclusions.

Zheng et al.,^27^ reviewed the efficacy of different treatment methods on obstruction caused by precipitated medication or lipids in CVADs. The review included seven studies (*n* = 130 participants). The results of the meta-analysis^27^ found intravenous perfusion of sodium hydroxide the most effective treatment for restoring patency (0.77, 95% CI [0.55, 0.92]), however, the authors noted the poor quality of the evidence should be considered when interpreting the result.

Overall, the results of focus area three indicated that most studies were either unable to draw a firm conclusion as to whether interventions such as heparin, anti-thrombotic or anti-clotting solutions, or flush technique were effective at reducing the risk of occlusion or restoring patency due to inadequate results or poor study quality as a result of bias, methodology or insufficient sample size. Only three of the included systematic reviews favoured the experimental intervention of the 10 analysed studies. Four were unable to draw a conclusion.

## Discussion

The general results of this review indicate that there is some evidence indicating that use of alternative solutions such as heparin, urokinase, sodium hydroxide, etc. or flushing technique may have some benefit on reducing CVAD occlusion rates or restoring patency, however several reviews were unable to determine this conclusively. Most reviews reported being impacted by the methodological quality or bias in their included studies which affected the certainty of their results. The most promising results were identified in the first focus area which covered training for nursing staff which identified that education or training for nursing staff (and healthcare professionals) appeared to have an overall positive effect on minimising CVAD-related complications like occlusions. Limiting occlusion risk and maintaining CVAD patency is a priority nursing intervention.^28^ Since nurses represent the majority of healthcare professionals and spend the greatest amount of time with patients, their decisions greatly influence patient management.^29^ Limiting occlusion risk and maintaining CVAD patency is a priority nursing intervention.^28^ Appropriate staff training supported by evidence-based practice is essential for an effective outcome. The right staff training is essential to deliver an outcome that is supported by evidence-based practice. As evidence in a quasi-experimental study conducted by Kelly et al.,^30^ staff confidence and competence can be raised through CVAD care and maintenance training, and education delivered through specialised theoretical and practical workshops. Targeted education programmes that include a pre and post testing of knowledge (and improved knowledge post education delivery),^31,32^ face to face training supported by a university, possibly through post graduate studies and followed up with a blended learning approach by adding e-learning^33,34^ have been identified as having some impact at improving nursing education and decreasing occlusions and related infections in patients with CVADs. Capability and competence can be improved through CVAD training, and education delivered through specialised theoretical and practical workshops.^30^ This can be further enhanced when combined with an eLearning component.^34^ Targeted education sessions, such as individual or group in service education sessions, delivered multiple times throughout a set time period concentrating on maintenance and care should be made mandatory and integrated into programmes for educating nursing staff to improve the experiences of patients with CVADs.^35^ Providing continuing education and periodic reinforcement of nursing skills can lead to improved patient outcomes. The goal of any healthcare professional is improved patient outcomes,^36,37^ which are directly correlated with enhanced nursing practice to reduce the occurrence of CVAD occlusion. Education sessions which included the use of training videos which focused on patient positioning, assessment for mechanical obstructions and communication identified a significant (*p* < 0.001) reduction in catheter occlusions from 29% to 8.5% in a 6-month period.^23^ Additionally, the training video increased nursing perception of self-efficacy and knowledge about CVADs.^23^

The introduction of CVAD specialist teams for device insertion and maintenance, according to Carr et al.,^38^ could enhance the experiences of patients living with a CVAD by lowering occlusion rates. Herring^39^ argues that establishment of a dedicated specialist vascular access team is a key intervention to decrease CVAD occlusion occurrences. When specialised CVAD teams were used, Johnson et al.,^40^ found that costs were reduced while efficiency, quality of care, patient satisfaction and patient outcomes improved. It is understood, that due to frequency and recency of practice that not every healthcare professional will be able to maintain their competence,^41^ hence it may be appropriate to form devoted CVAD champions within the clinical setting.

## Conclusions

CVADs are widely used to facilitate the delivery of therapies to patients who require long term intravenous access however are not without risk of complications such as occlusions. Occlusions impact patient care delivery and health system economy through treatment delays, investigations and interventions required to assess and manage the occlusion. This review has shown that education programmes utilising a variety of teaching strategies to increase competence of staff managing CVADs appear promising in reducing occlusion rates, however more high-quality evidence is required in the form of a well conducted RCT to better establish this effect. The efficacy of various catheter types, flushing techniques and locking solutions remains inconclusive. Heterogeneity of research outcomes in the published literature, as well as low quality and biased studies contributes to limit the external validity of evidence in this area of research and practice.
